# Plant Derived Phytocompound, Embelin in CNS Disorders: A Systematic Review

**DOI:** 10.3389/fphar.2017.00076

**Published:** 2017-02-27

**Authors:** Uday P. Kundap, Saatheeyavaane Bhuvanendran, Yatinesh Kumari, Iekhsan Othman, Mohd. Farooq Shaikh

**Affiliations:** Neuropharmacology Research Laboratory, Jeffrey Cheah School of Medicine and Health Sciences, Monash University MalaysiaSelangor, Malaysia

**Keywords:** embelin, CNS disorders, neuropharmacology, neurodegenerative diseases, natural product

## Abstract

A Central nervous system (CNS) disease is the one which affects either the spinal cord or brain and causing neurological or psychiatric complications. During the nineteenth century, modern medicines have occupied the therapy for many ailments and are widely used these days. Herbal medicines have often maintained popularity for historical and cultural reasons and also considered safer as they originate from natural sources. Embelin is a plant-based benzoquinone which is the major active constituent of the fruits of *Embelia ribes* Burm. It is an Indo-Malaysian species, extensively used in various traditional medicine systems for treating various diseases. Several natural products including quinone derivatives, which are considered to possess better safety and efficacy profile, are known for their CNS related activity. The bright orange hydroxybenzoquinone embelin-rich fruits of *E. ribes* have become popular in ethnomedicine. The present systematic review summarizes the effects of embelin on central nervous system and related diseases. A PRISMA model for systematic review was utilized for search. Various electronic databases such as Pubmed, Springer, Scopus, ScienceDirect, and Google Scholar were searched between January 2000 and February 2016. Based on the search criteria for the literature, 13 qualified articles were selected and discussed in this review. The results of the report showed that there is a lack of translational research and not a single study was found in human. This report gives embelin a further way to be explored in clinical trials for its safety and efficacy.

## Introduction

Central nervous system (CNS) is an integral part of the nervous system. It consists of the brain and spinal cord, and are associated with a number of important actions of the body. A CNS disease can be defined as one which affects either the spinal cord (myelopathy) or brain (encephalopathy) or both. The etiology of CNS involves a number of factors, for example, structural defects, infections, trauma, autoimmune disorders, tumors, neurodegeneration, and others, which may lead to neurological or neuropsychiatric or neurodegenerative or neurodevelopment disorders (Cannas et al., 2002; Upadhyay, 2014). The prevalence of CNS diseases is at least two times higher in developing countries than developed countries. According to World Health Organization (WHO), traditional medicines have become a topic of global importance. In many developing countries, a large proportion of the population relies heavily on traditional healers and phytomedicine for primary health care requirements. Concurrently, many people in developed countries have begun to turn to alternative or complementary therapies, including medicinal herbs (World Health Organization, 1999; Saraf, 2012).

**Figure 1:**
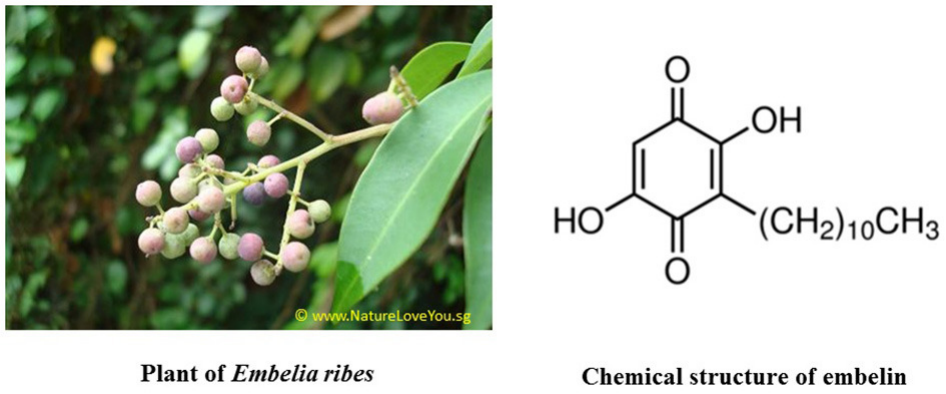
**Image of the plant *Embelia ribes* and chemical structure of embelin**.

Embelin is chemically known as 2,5-dihydroxy-3-undecyl-1,4-benzoquinone, which is the major active constituent of the fruits from *Embelia ribes* Burm (Family: Myrsinaceae), commonly known as “False Black Pepper” (Figure 1). It is an Indo-Malaysian species, reported from India, Sri Lanka, Singapore, Malaysia, and South China. *Embelia ribes* Burm is extensively used in Indian, Folk, Homeopathy, Tibetian, Unani, and Siddha traditional medicinal systems for treating various ailments like chronic inflammatory disorders, heart and urinary conditions, snake and insect bites, and tumor (Radhakrishnan et al., 2012). The dried fruit is considered anthelmintic, astringent, carminative, alterative, and stimulant (Nadkarni, 1996). Embelin is already studied for its safety and toxicity profile in rodents and non-rodents. It is reported that embelin is safe up to 3 g/kg orally when tested in rodents after acute exposure. Another report on subacute toxicity after repeated administration of embelin at 10 mg/kg dose found to be safe in rats (Poojari, 2014).

**Figure 2:**
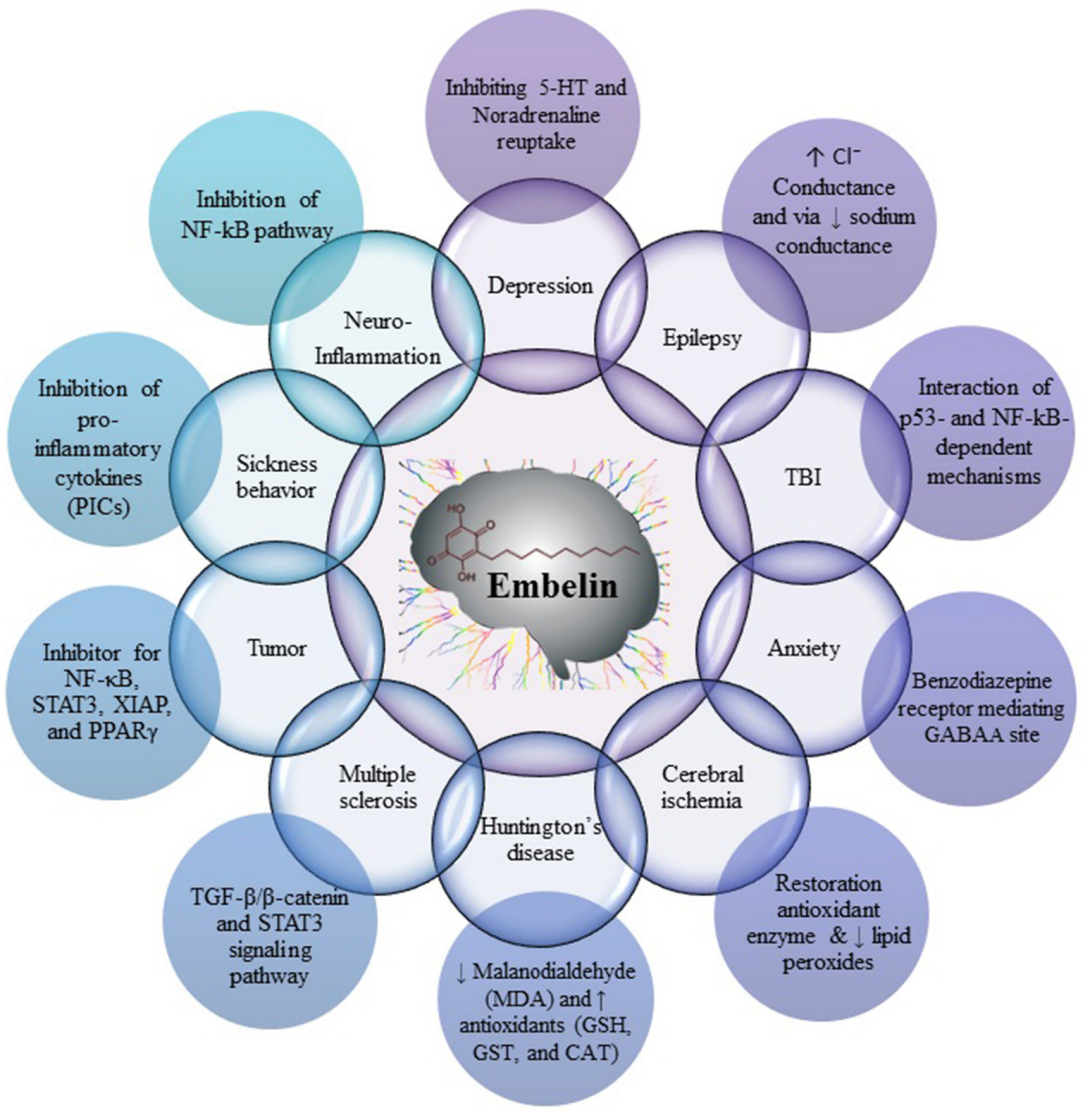
**Mechanism of action of embelin on various CNS disorders**.

Fruits of *E. ribes* have been used for the treatment of central nervous system (CNS) disorders, mental disorders and as a brain tonic in the traditional systems of medicine. Embelin was found to be useful in decreased cerebral infarction area and histopathological alteration, such as normal glial density, decreased edema, absence of lymphocytes, congestion of blood vessels, and necrosis. These reports suggest that embelin would be useful as an adjunct therapy for cerebral stroke and as a potent neuroprotective agent (Thippeswamy et al., 2011). Embelin posses all the characteristics of a compound which can cross the blood-brain barrier (BBB) and elicit an effect on the CNS (Pathan et al., 2009). Embelin reported for its CNS effect by diverse mechanisms, namely by scavenging free radicals and antioxidant effect, by inhibiting pro-inflammatory cytokines like NF-κB and p53, by modulating sodium channel, chloride conductance, and GABA_A_ receptor, by inhibiting STAT3, XIAP, and PPARγ pathways (Figure 2).

Embelin has been explored and reported for various CNS disorders using cell lines and animal models. There is no single study which summarizes the effectiveness of embelin in CNS associated disorders. Although embelin proved to be effective in laboratories against various CNS disorders, but it is not being translated to humans yet. In the present systematic review, an effort is being made to systematically review all the literature available with embelin in animal and clinical research.

## Materials and methods

### Search technique

The extensive literature search was done to conduct a systematic review summarizing the effects of embelin on central nervous system and related diseases. Various electronic databases were used, namely Pubmed, Springer, Scopus, ScienceDirect, and Google Scholar between the period January 2000 and February 2016. The following keywords were searched individually and in combination with the embelin: brain, trauma, CNS, neurological disorder, neurodegenerative disease, and psychological disorder.

### Study selection and exclusion/inclusion criteria

The search was limited to, articles published in English language and original research articles only. Abstracts of symposiums and conferences, review articles, books, and patents were excluded due to insufficient information for evaluation and comparison. Articles which were not related to CNS diseases were excluded. Any clinical, pre-clinical, *ex-vivo*, and *in-vitro* studies were also the part of the inclusion criteria.

### Data extraction

Two separate researchers obtain data independently, and then the titles and abstracts of each article were compared to delete duplication of the data. Based on the mentioned eligibility criteria for the literature search, 14 articles were excluded and 13 qualified articles were evaluated in this study. The aim of using PRISMA statement is to help authors to understand and improve the reporting of systematic reviews and meta-analyses related to use of embelin in CNS related disorders (Moher et al., 2015). Flow diagram was prepared according to the guidelines of PRISMA-Transparent reporting of systematic reviews and meta-analyses (Moher et al., 2009).

## Results and discussion

**Figure 3:**
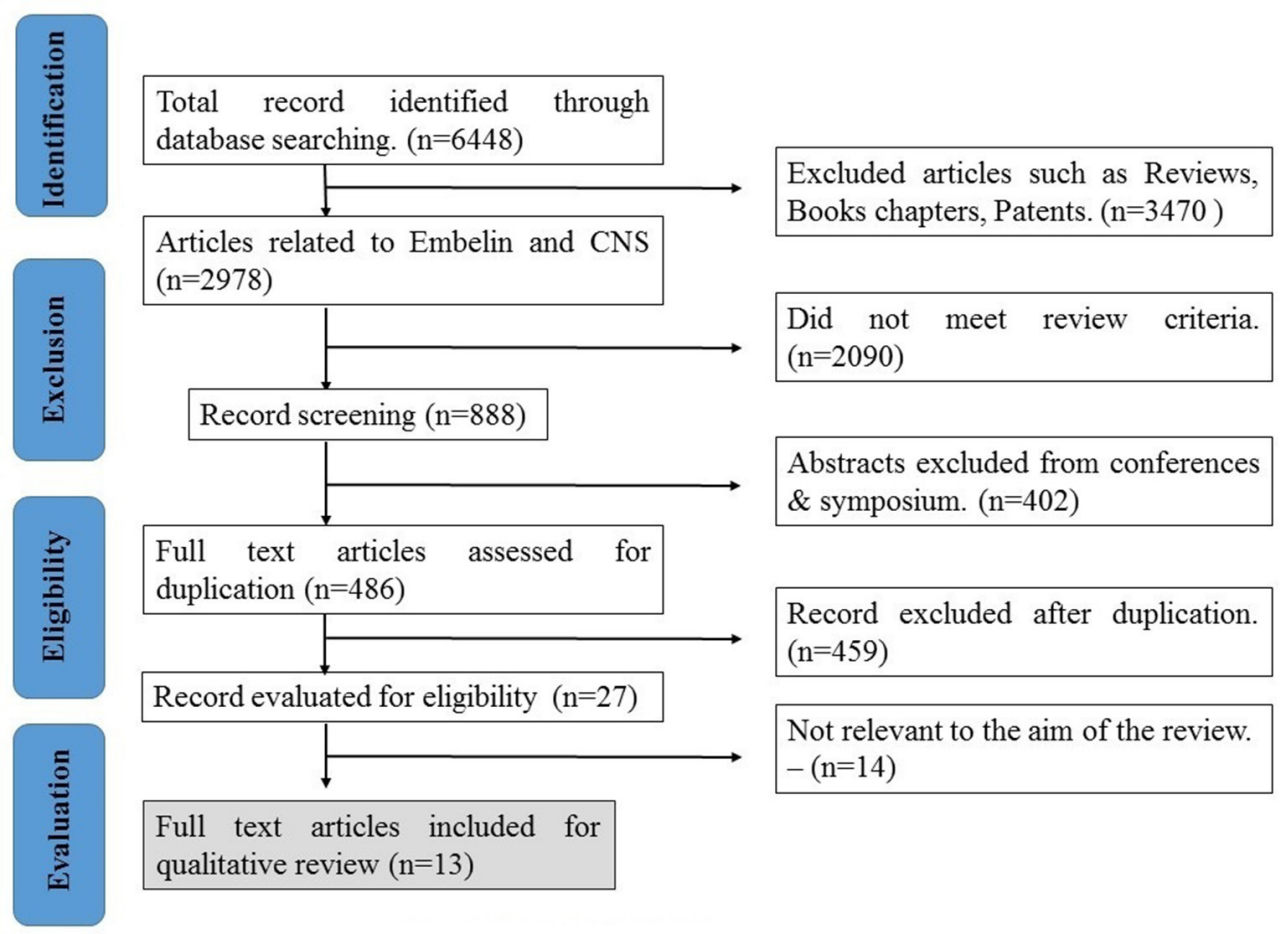
**Flow chart of study selection procedure**.

The search based on the keywords mentioned in the methodology yielded 6,448 records. After applying exclusion criteria, total articles removed were 6,435, which includes; (a) 3,470 reviews, book and patents, (b) 2,090 did not meet review criteria, (c) 402 abstracts, (d) 459 duplicates, and (e) 14 not relevant to the aim of the review based as they deal with formulations of the embelin (Figure 2). Thirteen eligible articles were included, compiled in Table 1 and discussed in the present systematic review (Figure 3).

**Table 1:** **Pharmacological activities reported with embelin in central nervous system related disorders**.

**Sr. no**.	**CNS activity**	**Study sample**	**Source of embelin**	**Dose**	**Result**	**Number of citations**	**Author (year)**
1	Anticonvulsant activity	Swiss albino rats (150–200 g; *n* = 6) Swiss albino mice (20–25 g; *n* = 6)	Embelin isolated from berries of *Embelia ribes*	2.5, 5, and 10 mg/kg	↓ in the duration of HLTE in MES (2.5 and 5 mg/kg, i.p.). Electroshock—100% protection against mortality. ↑ Clonic and tonic onsets at all dose.	54	Mahendran et al., 2011b
2	Antidepressant activity	Swiss albino mice (20–25 g; *n* = 6)	Embelin isolated from berries of *Embelia ribes*	2.5 and 5 mg/kg	Antidepressant-like effect in Tail suspension test (TST). ↓ Immobility in the Forced swimming test (FST). Exhibited significant activity in mice TST and FST experimental models.	6	Gupta et al., 2013
3	Anxiolytic activity	Swiss albino mice (*n* = 6)	Embelin isolated from berries of *Embelia ribes*	2.5 and 5 mg/kg	↑ Time spent and number of entries in open arm (elevated plus maze). ↓Decrease in the duration of immobility in light box (light and dark model). ↑Increase rearing assisted rearing and number of square crossed (open field test). Embelin showed its anxiolytic effect in dose-dependent manner.	6	Afzal et al., 2012
4	Sickness behavior	Male Swiss albino mice (25–30 g; *n* = 8)	Embelin isolated from berries of *Embelia ribes*	10 and 20 mg/kg	Embelin prevented anhedonia, anorexia. Ameliorated brain oxidative stress markers. Protective effect of embelin in LPS-induced sickness behavior in mice.	0	Shaikh et al., 2016
5	Huntington's disease	Adult Wistar rats (190–220 g; *n* = 8)	Embelin isolated from berries of *Embelia ribes*	10 and 20 mg/kg/day	Loss of body weight. Decreased the oxidative stress. Decrease of 69–76% brain lesion. Protect the neurons from 3-NP toxicity.	1	Dhadde et al., 2016
6	Multiple sclerosis (Autoimmune encephalomyelitis, CNS inflammation)	Female C57BL/6 mice, aged 6–8 weeks (*n* = 6)	Embelin pure form	25 and 50 mg/kg	·↓ Human CD14+ monocyte-derived dendritic cell differentiation. ·↓Duction in the EAE (experimental autoimmune encephalomyelitis) clinical score. ·↓ Inflammatory Th1 and Th17 cells in EAE.	9	Xue et al., 2014
7	Traumatic brain injury	Female Sprague–Dawley rats, male C57BL/6 mice(*n* = 10)	Embelin pure form	200 nM	Inhibition of NF-κB expression of XIAP increases in PFT-treated animals. p53 and NF-κB dependent mechanisms delayed neurodegeneration	62	Plesnila et al., 2007
8	Hypoxia-ischemia (HI) induced neurological injury	Female and male Wistar rats pups (*n* = 15)	Embelin pure form (Sigma-Aldrich, USA)	20 mg/kg embelin	Confirm sex differences in behavioral and anatomical outcome. XIAP acts to protect the female brain from the early HI injury.	18	Hill et al., 2011
9	Global ischemia/reperfusion-induced brain injury	Male Wistar rats (200–260g; *n* = 6)	Extraction of embelin from *Embelia ribes*	25 and 50 mg/kg	↑ Locomotor activity and hanging latency time. ↓ Beam walking latency. ↓ Lipid peroxidation. ↑ Total thiol content and glutathione-S-transferase neuroprotective agent and useful in the treatment of stroke.	22	Thippeswamy et al., 2011
10	Focal cerebral ischemia brain	Male Wistar rats (200-250 g; *n* = 6)	Embelin isolated from berries of *Embelia ribes*	50, 75, 100 mg/kg	Decreased the infarction and edema (100 mg/kg). Decreased MDA level (75 and 100 mg/kg). ↑ SOD and CAT (100 mg/kg).	0	Patel and Gohil, 2014
11	Cerebral ischemia	C57BL/6 male, GI female, and Ovx female mice(*n* = 7)	Embelin pure form (Sigma-Aldrich, USA)	20 mg/kg	Inhibitor of XIAP exacerbated stroke-induced injury in females but had no effect in males.	97	Siegel et al., 2011
12	Apoptosis in human glioma cells via NF-κB inhibition	Human glioma cell lines T98G, U87MG, and H4. Immortalized primary human fetal astrocytes (IM-PHFA)	Embelin pure form (Sigma-Aldrich, USA)	(0–50 μM)	Embelin suppressed proliferation of human glioma cells. Apoptosis in human glioma cells by inhibiting NF-κB. ↓ NF-κB activity by reducing nuclear translocation of p65.	20	Park et al., 2013
13	Apoptosis in human glioma cells via the mitochondrial pathway	Human brain glioma U87 cells	Embelin pure form (Sigma-Aldrich, USA)	(0, 50, and 100 μg/ml)	Time- and dose-dependent apoptosis of brain glioma cells. Arrest the cell cycle in the G0/G1 phase. Changes in brain glioma cell mitochondrial membrane potential. Shifting of Bax and Bcl-2 to cause apoptosis.	6	Wang et al., 2013

### Anticonvulsant activity

Mahendran et al. (2011b) isolated embelin from the berries of *E. ribes* and reported on the anticonvulsant activity of the embelin using maximal electroshock (MES) and pentylenetetrazole (PTZ). MES cause the spread of seizure similar to grandmal epilepsy. In MES method, brief high-intensity shock is applied to the head through corneal or ear electrodes with a stimulator that either delivers a constant current to constant voltage at a frequency of 50–60/s. The MES convulsions are divided into five phases such as the phase of tonic limb flexion, the phase of tonic limb extension, the phase of clonic convulsions, stupor, and recovery or death (Castel-Branco et al., 2009). Protection against hind leg tonic extension (HLTE) in MES predicts the ability of embelin to prevent the spread of seizure discharge from the epileptic focus in the brain and suppressing generalized tonic–clonic and partial seizures. Phenytoin is said to protect against seizures by causing blockage of voltage-dependent, voltage-gated sodium channels. This block sustains repetitive high-frequency firing of action potentials. The results show that there is a complete absence of HLTE in MES model when treated with embelin 10 mg/kg dose in comparison with phenytoin. It shows that embelin at 10 mg/kg dose might act via blockade of voltage-gated sodium channels. Embelin at 2.5, 5, and 10 mg/kg shows, dose-dependent activity against MES model which act of sustaining repetitive high-frequency firing of action potential exhibiting anticonvulsant activity. Pentylenetetrazole (PTZ) is used to induce clones seizure-like behavior with increased locomotor activity. The increased chloride conductance drives the membrane potential toward the reversal potential of the Cl↓ ion which is about –65 mV in neurons, inhibiting the firing of new action potentials. This mechanism is responsible for the anti-epileptic effects of GABA_A_ allosteric agonists. Embelin might increase chloride conductance which drives membrane potential, inhibiting the firing of action potential delaying onset of a clonic-tonic seizure in PTZ induced epilepsy. PTZ basically acts at the picrotoxin site of the GABA_A_ receptor and reduces chloride conductance which further leads to glutamate excitation (Desmond et al., 2012).

For each seizure model, embelin was administered intraperitoneally 30 min prior to the induction of MES and PTZ. Administration of embelin (2.5 and 5 mg/kg, i.p.) showed significant (*P* < 0.001) reduction in the duration of HLTE compared to the control. Based on the results, the study demonstrated that embelin at 10 mg/kg dose could significantly reduce the duration of the HLTE in MES model. Embelin at all the three doses significantly (*P* < 0.001) decreased the onset of stupor when compared to the control. Eventually, the percentage protection reported was 100% as no mortality was observed in all the embelin treated rats when challenged with maximum electroshock. On the other hand, embelin significantly delayed the onset of clonic and tonic seizures with an increased in a survival rate in a dose-dependent manner when checked against PTZ. Embelin also exhibited significant and dose-dependent delayed the onset of clonic-tonic actions and protection from PTZ induced mortality. At 5 and 10 mg/kg doses, it exhibited 50 and 83.33% protection against mortality. This study does not include the effectiveness of embelin in chronic models of epilepsy like lithium-Pilocarpine, kindling, or intracerebroventricular kainic acid. Based on the preliminary results, Mahendran et al. (2011a) postulated that embelin is a potent anticonvulsant phytocompound and the plausible mechanism is through GABAergic modulation. But, there was no supporting information like brain GABA estimation or GABA receptor expression were included in the published report.

### Antidepressant activity

Depression is one of the common neuropsychiatric disorder which contribute to the global burden of the diseases affects about 1 in 20 people across the world (Currie and World Health Organization Regional Office for Europe, 2000). Gupta et al. (2013) reported the anti-depressant effect of embelin in experimental animals using two universally accepted experimental models: mice tail suspension test (TST) and forced swimming test (FST). Embelin was isolated from fresh fruits of *E. ribes*. The TST is used as an experimental method in scientific research to measure stress in rodents. The FST—in a rodent is used for evaluation of the antidepressant efficacy of new compounds, antidepressant drugs, and experimental development that are aimed at translating or preventing depressive-like states. It is based on the observation that if an animal is subjected to short term inescapable stress then it will become immobile. It has been described as rendering a situation in which “behavioral despair” is induced; that is, the animal loses hope to escape the stressful environment. It is well-known that compounds which selectively bind to high-affinity benzodiazepine receptors possess both anxiolytic and antidepressant effects. The anxiolytic effect of embelin was shown to be mediated through the effect on the GABA system. The similar mechanism of antidepressant action cannot be ruled out (Afzal et al., 2012). It is, therefore, reasonable to assume that the observed antidepressant-like activity of embelin could be attributed to its known antioxidant effect. GABA_A_ receptors are allosteric modulatory sites for benzodiazepine. They are probably composed of five protein subunits, at least some of which belong to different subunit classes. So far GABA_A_ receptors have been identified as six alpha–four beta–three gamma-, and delta- and two-rho = *p* subunits. A 3D-structural model similarity, further shows that embelin is closely related with the well-known antioxidant alpha-tocopherol (AT, vitamin E), especially in the long-chain non-polar tails and polar phenolic heads (Lobato et al., 2010). Embelin at 2.5 and 5 mg/kg dose administered orally and tested using tail suspension test (TST) and forced swimming test (FST) in mice. It was found to effectively reduce immobile time in both experimental models suggesting its antidepressant potential. Embelin (5 mg/kg) was reported to be comparably better than the standard antidepressant drug, imipramine (15 mg/kg), which is a tricyclic antidepressant drug (Gupta et al., 2013). As a criticism, Gupta et al. (2013) fail to mention about actual activity of embelin as an anti-depressant, which may be via inhibiting 5-hydroxytryptamine receptors (5-HT) and noradrenaline (NA) reuptake. In their study, imipramine was used as a positive control which has the known antidepressant activity and it acts by inhibiting NA and 5-HT reuptake into neurons.

### Anxiolytic activity

Anxiety is a feeling of discontent, such as fear or worry that can be intense or gentle. Everyone has feelings of anxiety at some point in their life for example, you may feel worried and anxious about a job interview or having a medical examination or sitting an exam. An anxiolytic is a medication or other intervention that inhibits anxiety. This effect is in contrast to anxiogenic agents, which increases anxiety. Afzal et al. (2012) revealed the anxiolytic potential of embelin using behavioral models of anxiety. The elevated plus maze (EPM) is a test used to measure anxiety in laboratory animals. The test uses two open and two enclosed arm apparatus with an elevated, plus-shaped (+). The behavioral model is based on the general disinclination of rodents to open spaces. This disinclination leads to the behavior termed thigmotaxis, a greater liking to remain in enclosed spaces or close to the edges of a bounded space. Reduction in anxiety is indicated in the plus-maze by an increase in the amount of time spent or entries in the open arms (time or entries in open arms/total time or total entries in open or closed arms; Walf and Frye, 2007). Open field test (OFT) is an experiment used to assay general locomotor activity levels and anxiety in rodents. Rodents display a natural aversion to brightly light areas. They also have an urge to explore a perceived threatening stimulus. The result of these two conflicting drives is anxiety. The increase in exploratory behavior leads to decreased anxiety. Increased anxiety will result in less locomotor motion and the animal will have a preference to remain at the edges of the field (Ramos, 2008). The light/dark test is based on the natural version of rodents to brightly illuminated areas and on the spontaneous exploratory behavior of rodents in response to mild stress, that is, novel environment and light. The test apparatus consists of a small dark safe compartment (one-third) and a large illuminated preference compartment (two-thirds; Bourin and Hascoët, 2003). Embelin at 5 mg/kg dose significantly increased the percentage of time spent and the number of entries in open arm in EPM apparatus. Percentage of time spent in the open arms and number of open arm entries was significantly (*P* < 0.01 and *P* < 0.001) increased by embelin (2.5 and 5 mg/kg) and diazepam. Time spent in the open arm by animal treated with embelin 2.5 and 5 mg/kg dose was 47.92 ± 1.25 and 66.17 ± 1.93 and no. of entries in the open arm by animal treated with embelin 2.5 and 5 mg/kg dose was 5.61 ± 0.47 and 7.90 ± 0.45 significant. The result shows that embelin exhibited dose-dependent activity as an anxiolytic in mice EPM-test. In the open field test, embelin exhibited a significant increase in a number of rearing, assisted rearing and number of the crossing. A number of rearing in open field test by animal treated with embelin 2.5 and 5 mg/kg dose was 17.68 ± 0.52 and 20.33 ± 0.59 and number of assisted rearing in open field test by animal treated with embelin 2.5 and 5 mg/kg dose was 16.20 ± 1.00 and 21.12 ± 1.2. In light and dark model, embelin produced a significant increase in time spent, the number of crossing and decrease in the duration of immobility in a light box. The animals treated with diazepam (1 mg/kg) and embelin (2.5 and 5 mg/kg) showed significant (*P* < 0.05 and *P* < 0.001) increase in the time spent in the lighted box and decrease in the time spent in the dark box. Time spent in the lighted box (s) by animal treated with embelin 2.5 and 5 mg/kg dose was 94.92 ± 1.73 and 116.8 ± 4.24 and time spent in a dark box (s) by animal treated with embelin 2.5 and 5 mg/kg dose was 167.0 ± 4.59 and 148.3 ± 1.26. Embelin at 2.5 mg/kg dose, failed to produce any significant change in the number of crossing and duration of immobility. Afzal et al. (2012) concluded that embelin exhibits significant anxiolytic activity in a dose dependant manner. They proposed that the observed activity could be due to an antagonistc effect on GABA receptor complex as most of the anxiolytic and antidepressant molecules selectively bind to high-affinity benzodiazepine binding site, present on GABA receptor. Both Gupta et al. (2013) and Afzal et al. (2012) contradicted about vehicle used to dissolve embelin, they mentioned two different vehicles, olive oil and 1% Tween 80 (v/v) as embelin has poor water solubility.

### Sickness behavior

During the course of an infection, the adaptive behavioral changes that develop in ill individuals is known as sickness behavior. It is relevant to understand depression and some aspects of the suffering in any disease. Sickness behavior is like a complex behavior induced by infections and immune trauma and mediated by pro-inflammatory cytokines. Some of the evidence state that sickness behavior is mediated through the effects of pro-inflammatory cytokines (PICs), such as IL-1, TNFα, and IL-6 (Maes et al., 2012). Embelin has been reported to possess neuroprotective, anxiolytic and antiinflammatory assets and has been shown to inhibit Nf-κB pathway and cytokine production (Mahendran et al., 2011a). Few characteristics of the behavioral pattern including malaise, hyperalgesia, pyrexia, listlessness, and disinterest in social interactions with the environment, lethargy, behavioral inhibition, exploration and grooming, reduction of reproductive performance, anorexia and weight loss, failure to concentrate, and anxiety (Maes et al., 2012). The effect of embelin was evaluated in sickness behavior in mice by Shaikh et al. (2016). Adult male Swiss albino mice were pre-treated with embelin (10 and 20 mg/kg per oral) for 3 days and then challenged with lipopolysaccharide (LPS; 400 μg/kg intraperitoneal). In EPM-test, pre-treatment with embelin (10 and 20 mg/kg) and dexamethasone (1 mg/kg) significantly reversed LPS-mediated effects and increased both the number of open arm entries (3.00 ± 0.53, 3.12 ± 0.58 and 3.12 ± 0.47, respectively) and time spent in open arm (15.38 ± 3.19, 14.00 ± 2.67 and 13.63 ± 1.94 s, respectively) when compared with LPS-alone. In light–dark box test, Pre-treatment with both the tested doses of embelin and dexamethasone (1 mg/kg) prior to LPS-shot significantly increased the time spent in the light compartment (33.88 ± 2.11, 43.75 ± 6.81 and 34.13 ± 4.38 s, respectively). In the forced swim test, embelin (10 and 20 mg/kg) prior to LPS-injection significantly decreased the floating time (78.75 ± 5.03 and 62.88 ± 5.03 s, respectively) when compared with LPS-alone-administered group. In social behavior tests, social exploration was measured just before the administration of LPS and again 2, 4, 8, and 24 h later. LPS-associated reduction in social behavior was attenuated by pre-treatment with embelin 10 mg/kg (20.61 ± 4.15%, 29.24 ± 8.45% and 56.61 ± 5.44%, respectively) and 20 mg/kg (38.41 ± 5.90%, 44.78 ± 5.17% and 63.55 ± 5.95%, respectively), dexamethasone 1 mg/kg (43.84 ± 5.31% 49.12 ± 2.95% and 64.87 ± 4.42%, respectively) when compared with LPS-alone-treated animals. In the open field test, pre-treatment with embelin (10 and 20 mg/kg) and dexamethasone (1 mg/kg) significantly attenuated LPS-induced changes and increased the peripheral, central and total number of line crossings and a number of climbs rear when compared with LPS-alone-treated group. Food and water intake test, pre-treatment of LPS-challenged mice with embelin (10 and 20 mg/kg) and dexamethasone (1 mg/kg) significantly reversed LPS-induced anorexia and adipsia in comparison to animals with LPS-alone-treated group. This all comparative finding eventually concluded that embelin is neuroprotective against LPS-induced sickness behavior in mice (Shaikh et al., 2016).

### Huntington's disease

Huntington's disease (HD) is a progressive neurodegenerative disorder associated with severe degeneration of basal ganglia neurons, which affects muscle coordination and leads to mental decline and behavioral symptoms. Systemic administration of 3-nitropropionic acid (3-NP), an inhibitor of the mitochondrial citric acid cycle, results in a progressive locomotor deterioration resembling that of HD. It differs mechanistically from excitotoxic lesions in that 3-NP irreversibly inhibits the mitochondrial citric acid cycle and leads to depressed ATP levels and elevated lactate concentrations (Borlongan et al., 1997; Brouillet, 2014). The study carried out by Dhadde et al. (2016) evaluated the neuroprotective potential of embelin against 3-nitropropionic acid (NP) induced experimental HD in rats. 3-NP significantly altered the behavioral and neuronal antioxidant status and caused significant neuronal damage in the striatal region. Elevated levels of malondialdehyde (MDA) and decreased levels of antioxidants (GSH, GST, and CAT) in the 3-NP treated rat brains supports the increased oxidative stress in HD. Behavioral tests were carried in the following order: neurological scoring, locomotor activity, EPM-test, beam walking test and hanging wire test. Biochemical estimation and brain lesion measurement were carried out in order to explore the molecular and structural differences of embelin in the brain. Administration of 3-NP alone shows motor abnormalities, decreased locomotor counts, loss of memory in EPM, decreased motor coordination in beam walking test, decreased hanging latency on hanging wire test and even 3-NP alone treatment resulted in highly significant (*p* < 0.001) reduction in body weight. In neurological scoring, none of the rats in embelin treated groups (10 and 20 mg/kg) showed hind limb paralysis and inability to move indicating its potent activity in reversing 3-NP induced motor abnormalities. The treatment with embelin at both the doses (10 and 20 mg/kg) reversed the decrease in locomotor counts induced by 3-NP toward the normal, and it was found to be 129.2 ± 5.58 and 160 ± 11.14, thus both the doses of embelin showed improvement in the locomotor count. In the EPM-test, embelin treatment at 10 mg/kg body weight significantly (*p* < 0.01) Reversed the memory loss (27.73 ± 3.92%) induced by 3-NP toward the normal, when compared with 3-NP alone treated animals. However, embelin at 20 mg/kg body weight dose showed a complete reversal of 3-NP induced memory loss same as a normal control group. At beam walking test, treatment dose of embelin (10 and 20 mg/kg) to 3-NP treated rats significantly (*p* < 0.001) improved the motor coordination and body balance. These animals traversed the beam in 5.11 ± 0.66 and 5.51 ± 0.72 s, respectively. In hanging wire test, embelin at doses of 10 and 20 mg/kg increased 3-NP induced decrease in hanging latency period, with values 36.66 ± 1.78 (*p* < 0.05) and 49.34 ± 2.62 s. The percentage decrease in the brain lesion area in both these groups was 69.59 and 76.21%, respectively. Embelin at 10 and 20 mg/kg to 3-NP treated animals significantly (*p* < 0.01) reduced the brain lesion area to 4.32 ± 0.44 and 3.38 ± 0.17%, respectively. Embelin treatment significantly protected neurons against 3-NP induced toxicity and reduced brain lesion up to 76%. It also exhibited a significant antioxidant and improved behavioral alterations induced by 3-NP. It is postulated that effectiveness of embelin could be due to its antioxidant potential and ability of embelin to modulate Ca^2+^ influx associated with increased brain glutamate levels (Dhadde et al., 2016). In 3-NP induced HD like condition model in rats, embelin found to be effective neuroprotectant.

### Multiple sclerosis (MS)

A chronic, typically progressive damage to the sheaths of nerve cells in the brain and spinal cord is termed as multiple sclerosis (MS). Symptoms may include numbness, impairment of speech and muscular coordination, blurred vision, and severe fatigue (Loma and Heyman, 2011). Animal models of brain inflammation are used to study autoimmune encephalomyelitis, or experimental allergic encephalomyelitis (EAE). Dendritic cells (DCs) have a pivotal role in the immune response and in stimulating naïve T-lymphocytes. Induction and maintenance of self-tolerance is a critical role of DCs and the failure of which can lead to autoimmune/inflammatory diseases. Embelin concentrations of 10, 30, and 60 μM, inhibits the differentiation and endocytosis of Human Monocyte-Derived dendritic cell (DCs). Compared with the day 5 untreated iDCs, a significant dose-dependent reduction in cell surface marker expression was observed in EB-treated cells. These results indicate that embelin inhibited the differentiation of human CD14^+^ monocytes into DCs in a dose-dependent manner. DC-derived cytokines are required for the polarization of the adaptive immune response. Therefore, Xue et al. (2014) investigated the potential effects of embelin on the regulation of the expression of the cell-polarizing cytokines. The production of the inflammatory cytokine tumor necrosis factor-alpha (TNF-α), the Th1 cell polarizing cytokine IL-12p35, the Th17 cell-polarizing cytokines IL-6 and IL-12/23p40, and the Th1 cytokine IFN-γ is substantially inhibited by embelin. Embelin suppressed the DC-mediated polarization of Th1 and Th17 cells and that it may be useful for the treatment of autoimmune inflammatory diseases that are mediated by Th1 and Th17 cells. Embelin ameliorates the clinical severity of experimental autoimmune encephalomyelitis (EAE). Compared with PBS-treated mice, the incidence of clinical symptoms in the 25 and 50 mg/kg/day EB-treated mice were reduced. These data suggest that embelin significantly ameliorates the clinical outcome of EAE. TGF-β/β-catenin and STAT3 signaling pathway are used by embelin to inhibit DC function, which leads to a reduction in the EAE clinical score and in CNS inflammation and demyelination. The novel finding of this study is that the anti-inflammatory effect of embelin appears to require the presence of functional TGF-β/β-catenin and the absence of activated STAT3 in DCs. It was also found that embelin-induced inhibition of the differentiation of Th1 and Th17 cells was associated with a down regulation of the production of Th1-polarizing and Th17-polarizing. Embelin, a novel XIAP inhibitor, significantly increased TGF-β/β-catenin signaling and decreased STAT3 phosphorylation in DCs (Xue et al., 2014).

Embelin is a potent inhibitor of the activation of pro-inflammatory transcription factors, such as nuclear factor kappa B and signal transducer and activator of transcription 3 (STAT3; Heo et al., 2011). Embelin has been shown to inhibit the X-linked inhibitor of apoptosis protein and various inflammatory pathways (Ahn et al., 2007). In one of the study, Xue et al. (2014) demonstrated that embelin possess a strong therapeutic potential for autoimmune inflammatory conditions in MS. The study revealed the role of embelin in modulating newer regulatory mechanisms and molecular targets essential for the effectiveness in EAE. Therefore, these reports suggest that embelin could be used as a therapeutic agent to control pathological conditions, such as MS and other inflammatory autoimmune diseases, that are induced by the functional expansion of Th1 and Th17 cells.

### Traumatic brain injury

Traumatic brain injury (TBI) is one of the common causes of mortality in both children and young adults. Survivors have many complications like brain edema and programmed death of neuronal cells following acute and chronic neurodegeneration. The study carried out by a team from five European institutes addresses the role and interaction of p53 and NF-κB-dependent mechanisms in TBI induced delayed neurodegeneration (Plesnila et al., 2007). Neuroprotection mediated by PFT is reversed by embelin in three different *in-vitro* models of neuronal cell death induced by camptothecin, glutamate, or oxygen-glucose deprivation (OGD). Embelin was used to evaluate whether enhanced X-chromosomal linked inhibitor of apoptosis (XIAP) levels is indeed involved in neuroprotection by pifithrin-a (PFT). Hence, they strongly suggest the involvement of NF-κB dependent regulation of XIAP in the observed neuroprotective effect (Plesnila et al., 2007).

### Hypoxia-ischemia (HI) induced neurological injury

Hypoxia-ischemia (HI) occurs when there is a deficiency in both oxygen and blood supply, which results in neonatal neurological impairment. Hill et al. (2011) tested on the caspase-dependent progression of apoptosis using embelin which is known as potent XIAP inhibitors in order to prove that sexes influences in differing pathways of cell death due to HI. So they found out that embelin inhibits XIAP by binding to BIR3 domain and thus eventually increase in cell death through a caspase-dependent pathway. Similarly, the behavioral outcomes showed that through XIAP inhibition, HI induced female rats possess severe behavioral deficits compared to HI males. These *in-vivo* data revealed that there were significant differences in severity of cognitive deficits in male infants compared to female infants with HI. This phenomenon supports the evidence of activation of caspase-independent cell death in males compared to females that activate caspase-dependent cascade following neonatal ischemia. By using embelin as XIAP inhibitor, they could conclude that gender influences cell death mechanism following HI injuries and suggest that it is very important to develop a sex-specific neuroprotection to cure HI.

### Ischemic stroke

The majority of strokes occur when blood vessels to the brain become narrowed or clogged with fatty deposits called plaque, This cuts off blood flow to brain cells. A stroke caused by lack of blood reaching part of the brain is called an ischemic stroke. Stroke is the third major cause of mortality and the leading cause of long-term disability. Ischemic stroke accounts for ~80% of all strokes (Jauch et al., 2013). Ischemic stroke can be divided into two main types: thrombotic and embolic. Deprived of oxygen and other nutrients, the brain suffers damage as a result of the stroke. A thrombotic stroke occurs when diseased or damaged cerebral arteries become blocked by the formation of a blood clot within the brain (Rha and Saver, 2007). In order to investigate the mechanisms underlying injury after ischemic stroke as well as to develop effective therapeutic approaches to the disease, several ischemic stroke models have been developed in a variety of species. Models of stroke that can be used in rodents are becoming increasingly popular at the bench because (1) genetically-engineered animals; (2) a number of neurosensory and motor behavior outcomes; (3) fewer animal welfare concerns. In general, there are four major types of animal models of ischemic stroke: (1) complete global cerebral ischemia; (2) incomplete global ischemia; (3) focal cerebral ischemia and (4) Multifocal cerebral ischemia (Liu and McCullough, 2011, Figure 4).

**Figure 4:**
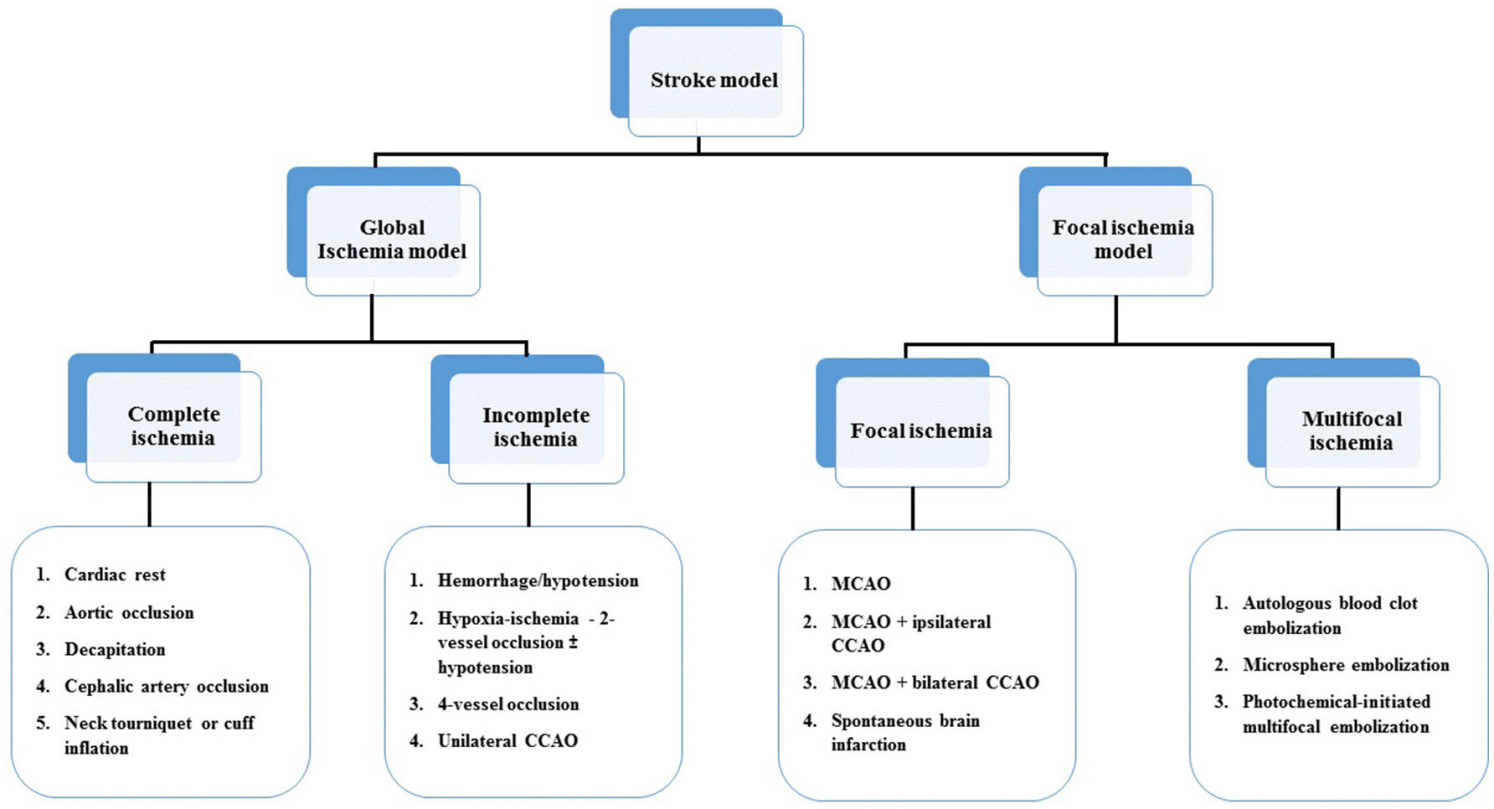
**Animal models of ischemic stroke model**.

#### Global ischemia

Brain tissue and cells require oxygen and nutrients to sustain survival and contribute to standard neural operating procedures which are deprived in the a global ischemic event. This leads to the death of brain tissue or cerebral infarction/ischemic stroke which is due to poor oxygen supply or cerebral hypoxia. Siegel et al. (2011) also hypothesized that caspase dependent mechanism of ischemic cell death is also influenced by gender differences. They found that XIAP mRNA level was higher in the normal female mice brain compared to stroke induced female mice whereas no differences were observed in the male brain. They reported that embelin decreased the association between XIAP and Caspase-3 in both sexes and was acting as an XIAP inhibitor. Based on the results, larger brain infarcts were seen in embelin treated ovariectomized (Ovx) females compared to gonadally intact (GI) females. The effects of embelin on infarct exacerbation may be due to independent of circulating estrogen levels. Siegel et al. (2011) concluded that embelin treatment significantly increases stroke-induced injury in females but had no effect in males. This shows that XIAP is an important mediator of sex-specific responses after stroke.

Thippeswamy et al. (2011) investigated the protective role of embelin in transient global ischemia induced by occluding bilateral common carotid arteries followed by reperfusion. Embelin pre-treated rats significantly improved locomotion. Vestibulomotor function was assessed by beam walking test and pre-treatment with embelin significantly decreased beam walking latency when compared with ischemic control animals. Grip strength was measured using Hanging Wire test and embelin treated animals had better and longer hanging time compared to ischemic control. The behavioral observations were very well-supported by biochemical estimations, where embelin found to be modulating the lipid peroxidation, the total thiol content and glutathione-S-transferase activity in brain homogenates. Histopathological studies confirmed, decrease in the infarct size in embelin-treated animals.

#### Focal cerebral ischemia

Cerebral ischemia is characterized by the inadequate oxygenated blood supply to the brain leads to the death of brain tissue It has been well-studied that reactive oxygen species play a key role in the pathogenesis of cerebral ischemia (Woodruff et al., 2011). Embelin is reported to have potent antioxidant activity and its chemical structure is similar to antioxidant coenzyme Q_10_ (Matthews et al., 1998). Patel and Gohil, investigated the effect of embelin against focal cerebral ischemia using the middle cerebral artery occlusion (MCAO) model. The model of MCAO involves the insertion of a surgical filament into the external carotid artery and threading it forward into the internal carotid artery (ICA) until the tip occludes the origin of the MCA, resulting in a cessation of blood flow and subsequent brain infarction in the MCA territory. Male Wistar rats were treated with embelin (50, 75, and 100 mg/kg, p.o.) for 20 days, followed by MCAO-induced focal cerebral ischemia and the parameters evaluated were infarct size and score. The antioxidant evaluation includes MDA, superoxide dismutase (SOD), and catalase (CAT) in brain homogenates. Embelin significantly decreased infarct size and improved infarct score. Embelin also decreased the MDA level whereas increased SOD and CAT level as compared to ischemic control group. The probable mechanisms by which embelin could be effective in cerebral ischemic condition is by the restoration of altered antioxidant enzyme activity as well as decreasing the production of lipid peroxides (Patel and Gohil, 2014). Some herbal medicines or their products having anti-oxidant activity have been suggested to protect against ischemic reperfusion injury, and thus justifying their use in cerebral ischemic patients.

### Brain cancer/apoptosis in human cancer cells

#### NF-κB inhibition

Glioblastoma is known to be the most aggressive primary brain malignancy with median survival rates (Chou et al., 2015). Embelin is an active compound that acts an inhibitor for NF-κB, STAT3, XIAP, and PPARγ to induce growth suppression and apoptosis in human cancer cells (Park et al., 2013). Embelin is a small-molecule inhibitor of an XIAP, which has the ability to specifically inhibit XIAP of various types of a tumor cell to control and regulate the apoptosis (Wang et al., 2013). A recent finding shows that embelin also enhanced TRAIL-mediated apoptosis Allensworth et al. (2012) and thus on the basis of above reference study, Park et al. (2013) suggested that embelin may be a good anti-cancer agent with less toxicity in normal cells. IκBs regulate nuclear translocation and activation of NF-κB, embelin decreases phosphorylation of IκBα in a dose- and a time-dependent manner, which indicates that embelin activates IκBα that is a negative regulator of NF-κB. In addition, furthermore decreased NF-κB activity as a transcriptional activator and they found that embelin reduced nuclear translocation of NF-κB. Embelin suppressed proliferation of human glioma cells without affecting the normal, immortalized human astrocytes. It also has been reported to induce apoptosis in human glioma cells by inhibiting NF-κB which plays an important role in cell proliferation and survival of tumor. However, embelin has found to show no inhibitory effect on XIAP in glioma cells, although this active compound was discovered as an XIAP inhibitor. Besides that, overexpression of p65 was decreased in embelin induced apoptosis glioma cells. So, they concluded that embelin could be a potent novel therapeutic compound by blocking cancer cell proliferation and inducing apoptosis through NF-κB inhibition.

#### Mitochondrial pathway

On the other hand, in support of the above finding, Wang et al. (2013) investigated the role of mitochondrial pathway played in embelin-induced brain glioma cell apoptosis and the effect of embelin on the cell cycle. Expression of apoptosis-associated proteins, Bcl-2, Bcl-xL, Bax, and Bak, as well as cytochrome-*c* levels, were determined by performing western blot analysis. Embelin was found to be apoptotic to brain glioma cells in a time and dose-dependent manner. The observed effect could be due to arrest of the cell cycle in the G0/G1 phase. Changes in mitochondrial membrane potential were caused by embelin in brain glioma cell. Additionally, embelin regulated the shifting of Bax and Bcl-2 to promote the mitochondrial release of cytochrome *c*, thus activating the caspase proteins to cause apoptosis. Thus, embelin induces apoptosis in brain glioma cells is closely associated with the mitochondrial pathway (Wang et al., 2013).

### Blood-brain barrier (BBB)—cerebral ischemia

Blood-brain barrier plays an important role in drug delivery to the CNS. Blood-brain barrier restricts, facilitates and regulates many substances from entering the CNS. It also secretes substances into the blood and the CNS (Banks, 2009). The entry of compounds across the BBB depends on their lipid solubility based on the estimation of oil/water partition coefficient (Laterra and Betz, 1999). Besides that, molecular weight, charge, tertiary structure and degree of protein binding are also among the factors in addition to lipid solubility affecting the ability of a drug to cross the BBB (Banks, 2009).

According to Pathan et al. (2009) a drug is likely to be able to transport across the BBB, if it possesses some important properties like, the compound should be in un-ionized form, partition coefficient (log P)-value should be near 2, molecular weight must be <400 Da and cumulative number of hydrogen bonds should not go beyond 8–10. According to this embelin is un-ionized molecule with log P-value of 4.83, the molecular weight is 294.38 and cumulative H bonds are 6. These properties of embelin make it permeable to BBB. So far, not a single study reported BBB permeability of embelin in *in-vitro* model. However, Siegel et al. (2011) performed *in-vivo* BBB permeability study and reported that embelin could cross the BBB. They performed liquid chromatography/tandem mass spectrometry (LC-MS/MS) on male and female sham and stroke brains. Embelin (20 mg/kg s.c.) was dosed for 3 days and it was found that the brain concentrations were elevated in both the sham and stroke mice, but the level was significantly higher in stroke mice which were close to reported IC_50_ for embelin (4.1 ± 1.1 μM).

### Safety and toxicity

Acute toxicity studies in mice treated with embelin 50 and 100 mg/kg oral dose showed no significant body weight change, mortality or apparent toxic effects, signifying its safety profile. This study suggests that embelin is safe on acute administration (Gupta et al., 1976). The LD50-value of embelin was reported as 44 mg/kg by i.p. route. Embelin in doses of 10 mg to 3 g/kg given orally to rats and mice did not show any toxic effects. Subacute toxicity on 10 weeks administration of 10 mg/kg of embelin to rats also indicated the drug to be free from toxic effects on heart, liver, kidney, and bone marrow, thereby having a high margin of safety in acute toxicity studies (Rathinam et al., 1976).

The toxicity of emblin has been assessed in female cyclic rats. Its administration at a dose of 120 mg/kg body weight did not cause any changes in the weight of liver, kidney, and spleen, however, the wet weight of the adrenals showed a remarkable increase. Biochemical constituents such as protein and glycogen did not show any change in these organs except in the adrenal where a significant increase was observed. The activity of acid and alkaline phosphatase was increased in the kidney and adrenal. These toxic effects seem to be due to exposure of a very high dose i.e., 120 mg/kg, whereas LD_50_ reported was around 44 mg/kg.

Administration of embelin for 6 weeks caused severe pathological changes in the liver and kidney which mainly included disintegration, necrotic changes, and perinuclear vacuolation. Marked tubular damage was observed in the kidneys. The adrenals showed hypertrophy and the histological features of the spleen remained unchanged (Prakash, 1994). In chronic toxicity study, the administration of embelin to Wistar rats at a dose of 50 mg/kg/day for 14 weeks did not cause any extreme drop in the blood counts but showed toxic effects on the hematopoietic cells (Sreepriya and Bali, 2006). Previous studies had also reported the non-toxic nature of embelin on hematopoietic cells when administered for 6 months in mice, rats, and monkeys (Radhakrishnan and Gnanamani, 2014).

For *in-vitro* cytotoxicity studies, embelin showed the toxic effect at 217 μg /ml to lung fibroblasts (Feresin et al., 2003). IC_50_ of 16.85 and 27.52 μM of embelin was calculated against mouse lymphocytes and mouse macrophages, respectively (Sreepriya and Bali, 2006). Isolated ovarian cells were directly challenged with embelin and showed a direct effect on isolated ovarian cells (Simukoko, 2000). It did not show the toxic effect on human fibroblasts at 20 μg/ml for 72 h in an *in-vitro* setting. Embelin was most active against sarcoma (XC) cells after 72 h of incubation (ED50 8 μg/ml) and slightly less active against Murine melanoma (B16) cells (ED50 13 μg/ml). An encouraging observation is a fact, that at these concentrations, embelin did not affect normal cells (HSF; Podolak et al., 2005).

Overall toxicity studies revealed that embelin at therapeutic doses found to be non-toxic and safe to use. Higher doses of embelin exhibit some sort of toxicity, but these doses are well above LD_50_-value and toxic effects are very much expected. There is also a need to carry out detailed toxicity study of embelin as per the International Council for Harmonization (ICH) safety guidelines.

## Conclusion and future directions

Embelin is the main constituent found in the plant *E. ribes*. Embelin posses favorable physical and chemical properties and its ability to cross the blood brain barrier make it a suitable candidate for the treatment of CNS disorders. In the present systematic review, an attempt was made to compile and discuss the efficacy of embelin against CNS complications. Embelin had been studied using various *in-vitro* prototypes and *in-vivo* animal models. It is well-reported that embelin exhibit strong anticonvulsant, anxiolytic, antidepressant properties and also improve conditions like sickness behavior, Huntington's disease, multiple sclerosis, cerebral ischemia and TBI.

Although a vast number of activities have been reported with embelin in experimental settings, there is not a single human study found on embelin related to CNS activity. None of the animal experimental outcomes was translated into human clinical research. One of the potential reasons for the non-translational research could be a lack of detailed safety and toxicity profile. Future pre-clinical and clinical trials are required to support the safety and efficacy of this active compound. Once safety profile is established, embelin should be taken up for clinical trials. As embelin is being studied for a rich number of CNS activities, a controlled human clinical trial will open up a new horizon for this promising molecule.

## Author contributions

UK and SB has equal contribution for first author. MS, UK, and SB contributed in perceiving and designing the study. UK and SB equally contributed with literature search and collection of data for the study. Data analysis and draft of the manuscript were completed by all authors. All the authors approved the content of the manuscript.

### Conflict of interest statement

The authors declare that the research was conducted in the absence of any commercial or financial relationships that could be construed as a potential conflict of interest.
